# Coming Late for Dinner: Localized Digestate Depot Fertilization for Extensive Cultivation of Marginal Soil With *Sida hermaphrodita*

**DOI:** 10.3389/fpls.2018.01095

**Published:** 2018-07-30

**Authors:** Moritz Nabel, Silvia D. Schrey, Hendrik Poorter, Robert Koller, Kerstin A. Nagel, Vicky M. Temperton, Charlotte C. Dietrich, Christoph Briese, Nicolai D. Jablonowski

**Affiliations:** ^1^Institute of Bio- and Geosciences, IBG-2: Plant Sciences, Forschungszentrum Jülich GmbH, Jülich, Germany; ^2^Institute of Ecology, Leuphana University Lüneburg, Lüneburg, Germany

**Keywords:** digestate fertilization, localized fertilizer placement, marginal substrate, perennial plants, rhizotron, root plasticity

## Abstract

Improving fertility of marginal soils for the sustainable production of biomass is a strategy for reducing land use conflicts between food and energy crops. Digestates can be used as fertilizer and for soil amelioration. In order to promote plant growth and reduce potential adverse effects on roots because of broadcast digestate fertilization, we propose to apply local digestate depots placed into the rhizosphere. We grew *Sida hermaphrodita* in large mesocosms outdoors for three growing seasons and in rhizotrons in the greenhouse for 3 months both filled with marginal substrate, including multiple sampling dates. We compared digestate broadcast application with digestate depot fertilization and a mineral fertilizer control. We show that depot fertilization promotes a deep reaching root system of *S. hermaphrodita* seedlings followed by the formation of a dense root cluster around the depot-fertilized zone, resulting in a fivefold increased biomass yield. Temporal adverse effects on root growth were linked to high initial concentrations of ammonium and nitrite in the rhizosphere in either fertilizer application, followed by a high biomass increase after its microbial conversion to nitrate. We conclude that digestate depot fertilization can contribute to an improved cultivation of perennial energy-crops on marginal soils.

## Introduction

Biomass is the primary source of energy in Europe’s renewable energy mix, contributing 67% to the total (Eurostat, 2017). However, if biomass (e.g., energy maize) for bioenergy purposes is produced on agricultural soils, it is competing with food crops for the same land resources, resulting in land use conflicts (FAO, 2009; Fritsche et al., 2010). The cultivation of energy crops on marginal soils is currently discussed as a more sustainable alternative, minimizing potential conflicts with food production (Schröder et al., 2008; Graham-Rowe, 2011). A sustainable use of marginal soils to produce biomass for bioenergy purposes requires adapted cropping strategies that give consideration to the specific soil conditions of marginal substrates and the environment with special attention to the biodiversity of plants and animals (Fang et al., 2007; Blanco-Canqui, 2010; Brüll, 2015).

Following the idea of a closed nutrient loop, organic residues such as biogas digestates are applied to the fields as organic fertilizers after the biomass conversion to bioenergy (Arthurson, 2009; Vaneeckhaute et al., 2013) thereby reducing the need for synthetically produced fertilizers (Haraldsen et al., 2011; Walsh et al., 2012). Further, the carbon content of such organic fertilizers can play an important role as soil amendment increasing the soil fertility of marginal substrates in the long-term (Beare et al., 1994; Tiessen et al., 1994; Reeves, 1997; Nabel et al., 2017). Combinations of perennial energy crops with organic fertilization have been discussed as a possible cropping scenario on marginal sites (Blanco-Canqui, 2010; Nabel et al., 2016). Perennial energy crops are of special interest for the cultivation of marginal soils, as their extensive and perennial root system increases their ability to access the limited water and nutrient resources, often characteristic for marginal soils (Voigt et al., 2012).

However, there are still obstacles that hold farmers off from bringing cropping systems based on organic fertilization, perennial plants and the use of marginal soils into agricultural practice. As first obstacle, physical soil properties like the low water-holding capacity of sandy marginal substrates may increase by surface application of organic fertilization such as digestates. On sandy soils, surface application of digestates strongly decreases the wettability of the substrate by increasing the water repellency of the sand via CH-groups, coating the sand grains, resulting in increased surface water runoff (Voelkner et al., 2015a,b). A second obstacle is the fact that digestates contain a high concentration of NH_4_^+^ (Möller and Müller, 2012), which can have a negative impact on the establishment of an extensive root system (Forde and Lorenzo, 2001; Nabel et al., 2014). As a third obstacle, sandy substrates with low water holding capacity (WHC) can be very prone to nitrogen leaching as nutrients are rapidly washed out of the rhizosphere (Di and Cameron, 2002; Nabel et al., 2016). Further risks for nitrogen losses are linked to the fact that digestates cannot be plowed into an established stand of perennial energy crops, because that would cause severe damage of the perennial root systems of the crops. Furthermore, the risk of nitrogen losses via gaseous NH_3_ and N_2_O emissions is high, irrespective of soil surface or subsurface application (Möller and Stinner, 2009; Rochette et al., 2009). In addition, perennial energy crops need special attention in the year of crop establishment, before they establish their extensive root system and become competitive against weeds (Borkowska et al., 2009). For the above-mentioned reasons broadcast digestate fertilization could counteract a fast and successful establishment of a vigorous and competitive perennial energy crop canopy.

In order to overcome these problems and allow for an efficient and sustainable use of digestates on sandy marginal substrates for plant biomass production, we aim to adapt the digestate fertilization to the Controlled Uptake Long Term Ammonium Nutrition (CULTAN) method proposed by Sommer (2003). According to this method developed for mineral NH_4_^+^-rich fertilizers, fertilizers are not applied broadcast but injected locally directly into the rhizosphere (Sommer, 2005). As the plant roots adjust to the heterogeneous distribution of nutrients in the rhizosphere, they can increase the rooting density and share of fine roots in the fertilized zone (Nkebiwe et al., 2016).

In recent years, the method has been adapted to organic fertilizers that are rich in NH_4_^+^ like sludge, manure slurries and digestates (Dell et al., 2000; Pote et al., 2011; Bittman et al., 2012). In various studies on agricultural soils planted with conventional agricultural crops or grasslands, a positive impact of a localized application of organic fertilizers on drought (Garwood and Williams, 1967; Ma et al., 2009), nitrate leaching (Baker, 2001), root growth and nutrient use efficiency (Hodge, 2004; Weligama et al., 2008) as well as NH_3_ volatilization (Nyord et al., 2008) was shown. All these positive effects of localized organic fertilizer placement sum up in increased biomass yields (Cooke, 1954; Jing et al., 2012). For more information about the underlying processes of the methodology we refer to the review by Nkebiwe et al. (2016), who also focused on localized placement of organic fertilizers into the rhizosphere.

The fertilizer placement technology, already well established on conventional agricultural sites, is however, not yet used for the application of digestates to perennial energy crop cultures grown on marginal sandy field sites. Still, the technology of controlled fertilizer placement tackles major problems of digestate fertilization on sandy marginal sites and is therefore of interest for investigation. In this study, we tested digestate fertilization, applied as localized nutrient depots in the rhizosphere of the perennial energy crop *Sida hermaphrodita*, grown on a sandy marginal substrate. We compared localized to a broadcast digestate fertilization and mineral fertilization using a conventional NPK-fertilizer and an unfertilized control. *S. hermaphrodita* is a prairie forb species from North America that grows well on sandy or rocky soils with low organic matter content, producing relatively high biomass yields even with low nutrient levels in the soil (Spooner et al., 1985; Borkowska et al., 2009). In order to follow the structural adaptation of the root system of *S. hermaphrodita* to the different fertilizer applications a rhizotron experiment was performed under controlled greenhouse conditions. To verify our results under conditions that resemble more the field conditions, we also conducted a 3-year outdoor mesocosm experiment where root proliferation of depot-fertilized plants were followed by using mini-rhizotrons.

The study was designed to answer the following research questions and hypotheses:

Question 1: What causes the reduced root growth on *S. hermaphrodita* seedlings following digestate application as fertilizer on marginal soil and how does it develop over time?
Hypothesis 1: The root growth is affected by the nitrogen turnover of the fertilizer. High concentrations of ammonium and nitrite following the fertilizer application instantly are toxic to plant roots. The mineralization of ammonium to nitrate will allow enhanced root growth at a later stage.
Question 2: What could be an effective measure to apply digestate fertilization and promote root growth at the same time?
Hypothesis 2: The application of localized patches of digestate (digestate depot) into the rhizosphere will promote root growth, as the major part of the rhizosphere remains unaffected by the fertilization and therefore by initial adverse effects on root growth.

## Materials and Methods

### Study Site and Plant Cultivation

A glasshouse rhizotron study and an outdoor mesocosm experiment were performed at Forschungszentrum Jülich GmbH, IBG-2: Plant Sciences, Germany (50°54′34″N 6°24′47″E). For the glasshouse experiment, 80 *Sida hermaphrodita* seedlings of BBCH stage 12-13 (Jablonowski et al., 2017) were transplanted in February 2016 to a rhizotron (inner dimensions: height: 75 cm; width: 36 cm; depth: 2.6 cm) filled with a sandy substrate (RBS GmbH, Inden, Germany; particle size: ≤1 mm; pH 6.6, no detectable amounts of N, P, K, and C), accounting for 80 rhizotrons in total. Rhizotrons were irrigated manually three times a week to 60–70% of substrate WHC. The detailed climate data for temperature and exposure to light over the 3 months experimental time are presented in **Table 1**.

**Table 1:** Climate data for the greenhouse rhizotron experiment and the outdoor mesocosm experiment: mean temperature, precipitation and daily light integral (DLI) values during the experimental time from 2014 to 2016 at the Forschungszentrum Jülich GmbH (50°53′47″ north and 6°25′32″ east; 80 m a.s.l.).

Year	Mean air temperature (°C)		DLI (mol m^−2^ day^−1^)	Precipitation (mm)
	Day	Night		
**Greenhouse rhizotron experiment**							
2016	22.0	16.0	7.4	–
**Outdoor mesocosm experiment**				
2014	15.2		37.0	801.4
2015	15.1		39.9	678.1
2016	15.8		37.7	651.3

An outdoor mesocosm experiment was conducted using 21 containers, each filled with 250 L of the same sandy substrate as used in the rhizotrons. Big outdoor containers were chosen to keep growing conditions of *S. hermaphrodita* as close to field conditions as possible (Poorter et al., 2012a, 2016). Seedlings of *S. hermaphrodita* of BBCH stage 13–14 were transplanted into the mesocosms in May 2014 (Jablonowski et al., 2017). The detailed establishment of *S. hermaphrodita* plants into the mesocosms was described earlier (Nabel et al., 2016). The climate data over the 3-year experimental time can be found in **Table 1**.

### Treatments and Fertilization

**FIGURE 1:**
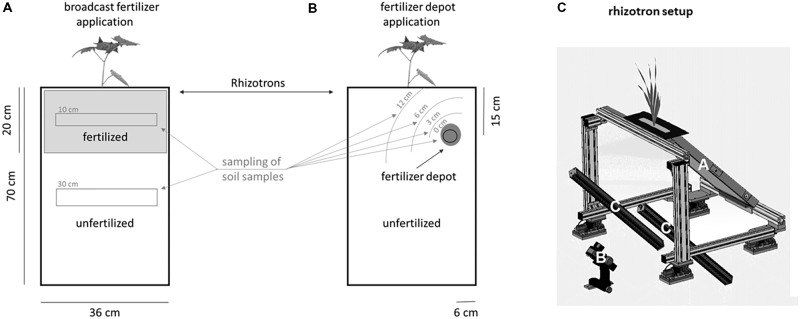
Illustration of the fertilizer application as broadcast (**A**: homogeneous digestate/NPK application) or depot (**B**: localized digestate application) fertilization in rhizotrons and soil sampling schema. Control rhizotrons did not receive any fertilization and are therefore not included in the illustration. **(C)** Schematic drawing of the imaging station of the automated phenotyping platform, *GrowScreen-Rhizo2*. The inclination angle of the rhizotron **(A)** is fixed to 45° with the transparent surface of the rhizotron facing downward. The image of the root system is taken by a high-resolution camera **(B)**, positioned at a distance of 1 m to the rhizotron surface. The positions and angles of the two light panels **(C)** are adjusted to allow homogeneous illumination and prevent reflections in the images.

Rhizotrons and mesocosms received digestate fertilization, applied either homogeneously incorporated (here after referred to as “broadcast fertilization”) or as a localized depot (here after referred to as “depot fertilization”) in the rhizosphere, broadcast mineral fertilization or no fertilizer supplement. The digestate was obtained from an operating biogas plant using maize silage as feedstock (digestate dry matter content: 7.2%; N_total_: 0.53%; NH_4_^+^: 0.32%; P: 0.14%; K: 0.68%; Mg 0.037%; Ca: 0.16%; S: 0.03%; organic matter: 5.3%, C:N ratio: 6; pH 8.2; all values referring to fresh weight; ADRW Naturpower GmbH and Co. Kg, Titz-Ameln, Germany). An NPK-fertilizer with an N:P:K-ratio similar to the digestate and a high share of ammonium was chosen to allow a comparison between the mineral and the organic digestate fertilization (NPK-fertilizer composition: N: 15% [1% nitrate; 9.5% ammonium; 4.5% isobutylidenediurea]; P: 5%; K: 8%; Mg: 3%; Compo Rasendünger, Compo GmbH, Münster, Germany). Both fertilizers were calculated to simulate a total N application of 40 t digestate ha^−1^, which was identified earlier as the optimum dose of digestate fertilization for *S. hermaphrodita* grown on sandy substrate (Nabel et al., 2014). In mesocosms fertilization treatments were applied in May 2014–2016 to the soil surface and immediately incorporated to minimize possible losses of nitrogen via NH_3_ (Hayashi et al., 2009). In rhizotrons, broadcast fertilization treatments were applied by homogeneously mixing fertilizers into the top 20 cm of the rhizotrons. For the localized digestate depot fertilization, the same amount of digestate was applied locally as a patch of 4.6 cm diameter. In mesocosms, the digestate depot was injected at a 20 cm distance from the shoot of *S. hermaphrodita,* at 30 cm depth. In rhizotrons, digestate depots were placed in 15 cm depth and 6 cm distance to the rhizotron wall (**Figure 1**). In rhizotrons each fertilization treatment was applied with 24 replicates (8 replicates × 3 harvests), the unfertilized control treatment was applied in eight replicates (only one harvest). In mesocosms all treatments were applied in six replicates (one harvest after each growing season).

### Measurements

Rhizotrons with *S. hermaphrodita* plants were placed into a novel phenotyping platform enabling simultaneous and non-destructive measurements of root and shoot growth of rhizotrons-grown plants (*GrowScreen-Rhizo2*, developed at Forschungszentrum Jülich GmbH, Germany). The design of the new phenotyping system *GrowScreen-Rhizo2* is based on the experiences with the *GrowScreen-Rhizo1* platform published in 2012 (Nagel et al., 2012). However, the *GrowScreen-Rhizo1* concept of having fixed positions for rhizotrons and a moving cabinet for imaging the rhizotrons has been changed to enable higher flexibility and modularity. In the new platform, rhizotrons are moved in trays (five rhizotrons per tray) on a conveyor system and transported to an imaging station which has a fixed position. The inclination angle of the rhizotrons is adjusted always to 45° with the transparent plate of the rhizotrons facing downward to force the roots to grow toward the transparent plate. Inside the imaging station individual rhizotrons are taken out of the tray and positioned automatically in front of a high resolution camera (AV GT6600, Allied Vision Technologies, Germany; resolution 142 μm per pixel) which is taking one image of the whole transparent rhizotron surface (**Figure 1C**). During image acquisition, the door of the imaging station is closed automatically with rolling cutter gates to prevent undesired light reflections on the Plexiglas plate of the rhizotrons and the roots are illuminated using two LED panels (30 W 40 mm × 40 mm × 940 mm, color temperature 4000 K, item Industrietechnik GmbH, Germany). After imaging, the gate is opened and the rhizotron is placed back to into the tray completing the routine. Pictures were taken automatically once a week. In addition, plants were harvested at 30, 60, and 90 days after transplanting seedlings to determine leaf, stem, and root biomass. For harvest, rhizotrons were opened and the sandy substrate was carefully washed out with tap water and roots were cleaned thoroughly. Leaves, stems, and roots were cut and separately dried at 70°C to constant weight to determine the biomass dry weight. The pictures of the roots, acquired by *GrowScreen-Rhizo2* during growth were evaluated for total visible root length and root distribution by using the software *GROWSCREEN-Root* in combination with a graphic tablet with pens (Wacom Cintiq 21UX, 31 CANCOM Deutschland GmbH, Düsseldorf, Germany) (Nagel et al., 2009). In order to visualize the spatial distribution of roots for any given treatment, we wrote an additional software to combine the data from all root images of each treatment in one image, representing root structure and distribution for any given treatment. To create these images, the area of a given root picture, as acquired by the *GrowScreen-Rhizo2* system, was subdivided into a grid of cells, sized 25 × 25 pixels each. Each cell was initialized with zero. Every time, any part of the root structure was found in a given cell, the value of this particular cell increased by one. Further limiting the maximum increase per pixel per plant to one, allowed us to visualize root distribution in relation (%) to the number of plants per treatment. The generated images were converted from gray scale single channel images to three channel false-color images (Ware, 1988). This conversion was done using Opencv’s build-in function applyColorMap using the JET colormap (Bradski, 2000).

In mesocosms, the above-ground biomass was harvested at the end of the growth season in 2014, 2015, and 2016, and dried at 70°C to constant weight to determine the biomass dry mass. In 2016, the root biomass of four replicates for each treatment was determined by washing out the substrate, cleaning roots thoroughly and drying them at 70°C to constant weight. Root growth of plants grown in mesocosms was monitored non-invasively by mini-rhizotrons, which are Plexiglas tubes horizontally installed in 30 and 60 cm depth of the mesocosms. Pictures of root systems were taken after 6, 18, and 30 months after planting, using the CI In-Situ Root Imager (CID Bio-Science Inc., Camas, WA, United States). The pictures were than further processed as described above by using the software *GROWSCREEN-Root* to determine root length and distribution.

Daily light integral values (DLI) were directly measured at place on the rhizotron and mesocosm facility, employing a LI-COR: Li-190 device (LI-COR Environmental – GmbH, Bad Homburg, Germany).

Soil samples were taken at the date of destructive biomass harvests in each rhizotron. In unfertilized-, mineral-fertilized and broadcast digestate-fertilized rhizotrons, one mixed sample was taken in a depth from 0 to 20 cm and a second in the depth of 25–50 cm. In rhizotrons with digestate depot fertilization, one sample was taken in a radius of 3, 6, and 12 cm around the digestate patch, in each rhizotron (**Figure 1**). Soil samples were stored at 4°C until determination of NH_4_^+^, NO_2_^−^, NO_3_^−^, electric conductivity and pH were performed. NH_4_^+^, NO_2_^−^, and NO_3_^−^ were measured via ion chromatography (Dionex DX-500, AS23; eluent: 0.8 mM sodium bicarbonate and 4.5 mM sodium carbonate) in 0.1 M KCl extraction. Soil pH was determined using standard electrodes (Hanna Instruments pH 209 pH-meter, Vöhringen, Germany), using 0.01 M CaCl_2_ solution at 20°C. Nitrogen content of *S. hermaphrodita* leaves was determined by elemental analysis (VarioELcube, Elementar Analysensysteme GmbH, Langenselbold, Germany) after milling dried leaves for 60 sec. in a ball mill at 30 Hz (Retsch Mixer Mill MM 400, Retsch GmbH, Haan, Germany).

### Statistical Analysis

The rhizotron experiment consisted of four treatments (digestate broadcast fertilization, localized digestate depot fertilization, mineral broadcast fertilization, and unfertilized control) with three sampling dates (30, 60, and 90 days), with eight repetitions per treatment. The mesocosm experiment had the same treatments in six repetitions of which the above ground biomass was harvested at the end of the vegetation period in 2014, 2015, and 2016. The below-ground biomass of the mesocosms was harvested (*n* = 4) for each treatment in October 2016. Statistical analysis was performed with analysis of variance (ANOVA) with an *a posteriori* test in R 3.0.3 (The R Foundation for Statistical Computing, 2014) using the work package “Agricolae” (de Mendiburu, 2014).

## Results

### Soil Analysis in Rhizotrons

Thirty days after transplanting the seedlings, all fertilization treatments showed a rapid conversion from ammonium to nitrate (**Table 2**). However, for the two digestate fertilized variants (broadcast and depot) also the intermediate product of mineralization, nitrite, was found in high concentrations, especially in a 3–6 cm radius around the digestate depot. In digestate broadcast fertilized rhizotrons, 85% of the measured nitrogen was found in the form of nitrate. In digestate depot fertilized rhizotrons, 20% of the measured nitrogen remained as ammonium and 33% was found as nitrite. Sixty days after planting, nitrite was not detected, while nitrate concentrations reached their maximum levels in the digestate-fertilized horizon of broadcast fertilized digestate rhizotrons as well as around the depot fertilized zones. Nitrogen in the form of ammonium was only detected in a 6–12 cm radius around the digestate depots. In digestate broadcast and NPK fertilized rhizotrons, only minor amounts of ammonium were detected in the fertilized horizon. Ninety days after planting, almost all nitrogen in all treatments was present in the form of nitrate, mainly located in the fertilized horizons of digestate broadcast -, NPK fertilization and a 3–6 cm radius around the digestate depots.

**Table 2:** Soil and leaf analysis of *S. hermaphrodita* grown in rhizotrons.

		Soil				Plant
	Depth/Radius (cm)	Ammonium ppm	Nitrite ppm	Nitrate ppm	pH	Leaf nitrogen %
**Day 30**						
Digestate broadcast (depth)	10	14.2 ± 3.5	1.5 ± 1.0	96.7 ± 33.1	6.4 ± 0.2	4.3 ± 0.6
	30	0 ± 0	0 ± 0	79.1 ± 31.0	7.0 ± 0.1	
Digestate depot (radius)	3	54.7 ± 10	92.5 ± 32.2	132.0 ± 45.0	8.5 ± 0.2	2.9 ± 0.3
	6	59.9 ± 7.1	86.4 ± 25.2	21.0 ± 11.7	6.4 ± 0.1	
	12	0 ± 0	5.4 ± 5.0	100.0 ± 51.8	7.1 ± 0.1	
NPK (depth)	10	37.3 ± 3.0	0 ± 0	138.6 ± 48.6	6.4 ± 0.1	4.3 ± 0.7
	30	0 ± 0	0 ± 0	58.6 ± 21.0	7.1 ± 0.1	
**Day 60**						
Digestate broadcast (depth)	10	5.1 ± 3.5	0 ± 0	250.4 ± 35.0	7.2 ± 0.0	4.9 ± 0.5
	30	0.1 ± 0.1	0 ± 0	3.1 ± 2.9	7.3 ± 0.0	
Digestate depot (radius)	3	0 ± 0	0 ± 0	187.9 ± 28.0	7.5 ± 0.1	4.1 ± 0.7
	6	91.8 ± 75.6	0 ± 0	161.0 ± 26.4	6.8 ± 0.0	
	12	78.3 ± 73.2	0 ± 0	54.4 ± 21.1	7.3 ± 0.1	
NPK (depth)	10	5.7 ± 1.9	0 ± 0	89.7 ± 15.4	6.8 ± 0.1	4.7 ± 0.4
	30	0 ± 0	0 ± 0	0 ± 0	7.3 ± 0.0	
**Day 90**						
Control (depth)	10	0 ± 0	0 ± 0	0 ± 0	7.3 ± 0.1	1.7 ± 0.2
	30	0 ± 0	0 ± 0	0 ± 0	7.3 ± 0.2	
Digestate broadcast (depth)	10	0 ± 0	1.8 ± 0.5	130.6 ± 34.1	7.0 ± 0.1	4.1 ± 0.4
	30	0.3 ± 0.2	0 ± 0	1.1 ± 1.0	7.2 ± 0.1	
Digestate depot (radius)	3	0 ± 0	0 ± 0	124.4 ± 18.2	7.1 ± 0.2	4.4 ± 0.5
	6	3.9 ± 1.4	0 ± 0	137.6 ± 16.9	6.6 ± 0.1	
	12	0 ± 0	1.3 ± 0.8	54.8 ± 14.8	7.0 ± 0.0	
NPK (depth)	10	0.1 ± 0.1	0 ± 0	110.4 ± 25.0	6.5 ± 0.1	4.1 ± 1.1
	30	0.2 ± 0.2	0.8 ± 0.5	0.0 ± 14.7	7.5 ± 0.1	

*Toxic nitrite concentrations occur especially after 30 days in a close radius to the digestate depot. All fertilization treatments were adjusted to a digestate application of 40 t ha^−1^. Digestate broadcast: homogeneously incorporated in the top 20 cm; Digestate depot: localized digestate application; NPK: homogeneously incorporated in the top 20 cm; Control: no fertilization. Rhizotrons n = 8 replicates for each treatment and sampling date; ± indicates the standard error.*

After 30 days, nitrogen fertilization, regardless of application technique, lowered the pH to 6.4. Contrastingly, the pH of the sandy substrate outside the 12 cm zone that was affected by depot fertilization had values of pH 7.0. Only within the radius closest to the digestate depots (<3 cm) pH 8.5 was reached. In the following 2 months, pH values generally increased and leveled off between pH 7 and 7.5. Lower pH values (6.6) were only found within a 6 cm radius around the digestate depots and the NPK fertilized horizon of mineral fertilized rhizotrons.

### Root Growth and Root System Architecture

#### Rhizotrons

**FIGURE 2:**
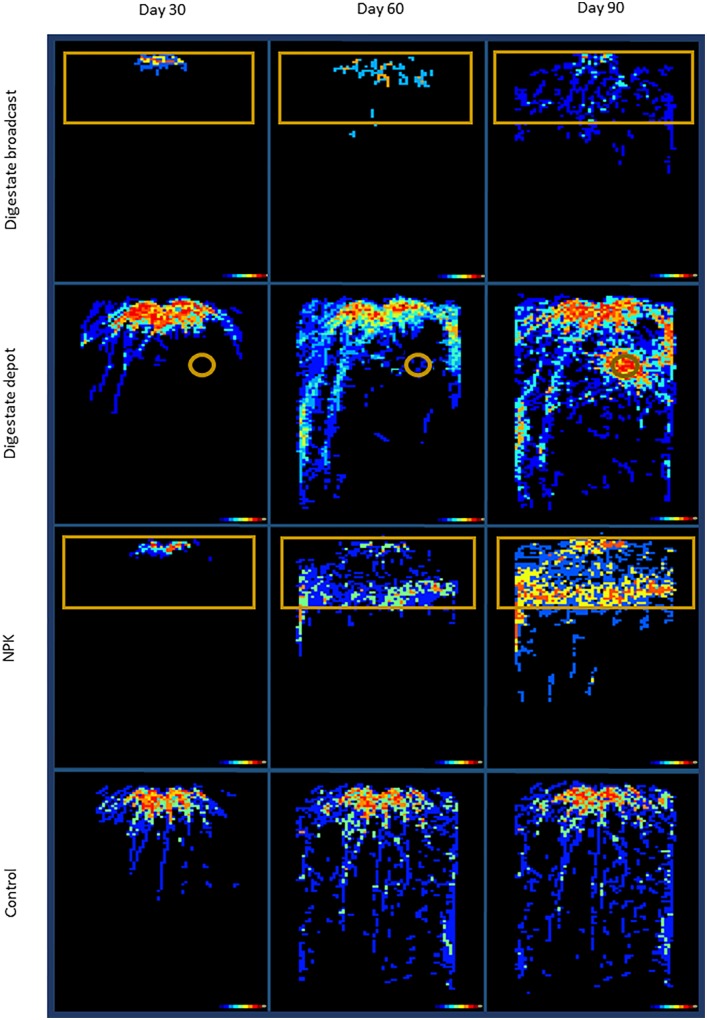
Hit-Map of rhizotrons. In digestate depot fertilized rhizotrons roots of *S. hermaphrodita* avoid the digestate depot zone for 60 days but form a dense root cluster in the depot zone after 90 days. Colors indicate the number of replicates that grew roots at the specific pixel (black: none; blue: 1–2; green 3–4; orange 5–6; red 7–8 replicates). Orange frames illustrate the fertilized zones of the rhizotrons. All fertilization treatments were adjusted to a digestate application of 40 t ha^−1^: Control: no fertilization; Digestate broadcast: homogeneously incorporated in the top 20 cm; Digestate depot: localized digestate application; NPK homogeneously incorporated in the top 20 cm; *n* = 8 replicates for each treatment and time of harvest.

**FIGURE 3:**
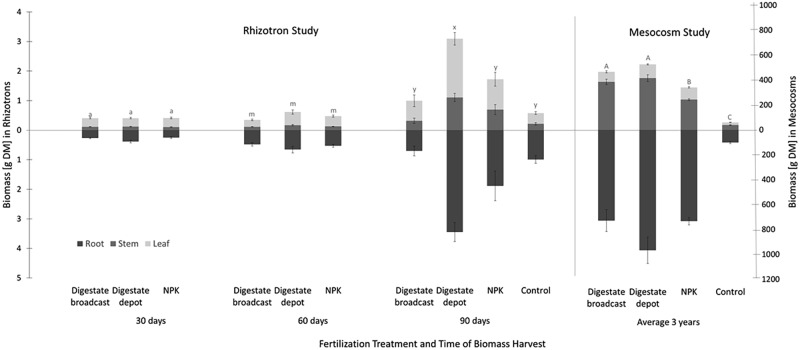
Plant biomass yield of *S. hermaphrodita* in rhizotrons and mesocosms. All fertilization treatments were adjusted to a digestate application of 40 t ha^−1^. Digestate broadcast: homogeneously incorporated in the top 20 cm; Digestate depot: localized digestate application; NPK: homogeneously incorporated in the top 20 cm; Control: no fertilization. Rhizotrons: *n* = 8 replicates for each treatment and time of harvest; Mesocosms: *n* = 7 replicates for each treatment harvested in 2014, 2015, and 2016. Root biomass was only measured in 2016. Values labeled with the same letter are not significantly different at *p* < 0.05; error bars indicate the standard error.

Thirty and sixty days after planting of *S. hermaphrodita* into rhizotrons, the form and application technique of the different fertilizers had a strong influence on the distribution of roots (**Figure 2**). However, root mass did not differ significantly between treatments until day 60 but differed strongly in their foraging behavior in the rhizotron experiment (**Figure 3**). Unfertilized control and digestate depot fertilized plants accessed the maximum depth of the rhizotrons within 60 days. Digestate broadcast and NPK fertilized plants only reached a rooting depth of 40 cm with 99% of the measured root length located in the fertilized horizon for digestate broadcast and 60 % for NPK fertilization, respectively. Digestate depot fertilization resulted in a strongly heterogeneous distribution of roots over the rhizotrons. Even though after 30 and 60 days when roots accessed deeper areas of the rhizotrons, no roots were present within a 6 cm radius around the digestate depots. After 60 days, only 1% of the total root length was located within this radius. After 90 days, the root growth pattern changed dramatically and 45% of the root biomass of digestate depot fertilized plants were found within this 6 cm radius around the digestate depots resulting in the formation of a dense root cluster around the digestate depot. Even after 90 days, roots in NPK and digestate broadcast fertilized rhizotrons did not reach the maximum depth of the rhizotrons and major part of their roots remained located in the fertilized horizon (60% for NPK and 80% for digestate broadcast fertilization).

#### Mesocosms

After 1 year of growth, *S. hermaphrodita* in mesocosms located 60% of the measured root length in a depth of 30 cm when fertilized with broadcast digestate or NPK-fertilizer. When fertilized with localized digestate depot, a root cluster was visible at 30 cm depth, accumulating 90% of the measured root length in 30 cm depth, while unfertilized control plants had 70% of the measured root length in the same depth. After 3 years root distribution generally shifted more toward the lower soil levels with 60% of the measured root length at 60 cm depth for digestate broadcast fertilized plants and 55% for NPK fertilized plants. Digestate depot fertilized plants as well as control plants still had the higher share of the measured root length in 30 cm depth with 55 and 60%, respectively.

### Biomass and Mass Fraction

#### Rhizotrons

**FIGURE 4:**
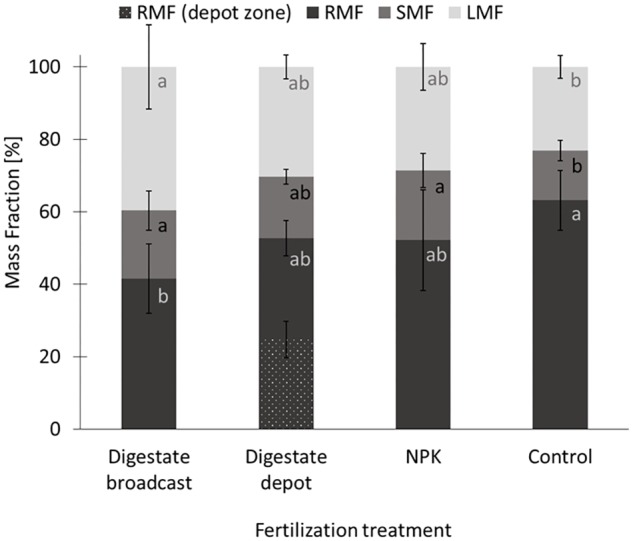
Mass fractions of *S. hermaphrodita* grown in rhizotrons for 90 days. RMF, root mass fraction; depot zone, zone in a 6 cm radius around the digestate depot; SMF, stem mass fraction; LMF, leaf mass fraction. All fertilization treatments were adjusted to a digestate application of 40 t ha^−1^. Digestate broadcast: homogeneously incorporated in the top 20 cm; Digestate depot: localized digestate application; NPK: homogeneously incorporated in the top 20 cm; Control: no fertilization; *n* = 8 replicates for each treatment. Values labeled with the same letter are not significantly different at *p* < 0.05. Error bars indicate the standard error.

In rhizotrons biomass increased on average by 40% from 0.7 g after 1 month to 1 g after 2 months, with no significant difference between treatments (**Figure 3**). However, after 90 days the biomass of digestate depot fertilized plants had increased by a factor 5–6.5 g. Broadcast fertilization with digestate or NPK did not result in a significant increase in biomass compared to the unfertilized control. At that time, unfertilized control plants as well as NPK and digestate depot fertilized plants accumulated approx. 50% of their total biomass in roots. Further, for digestate depot fertilized plants, half of the root biomass was located within a 6 cm radius around the digestate depot (**Figure 4**). Plants that received digestate broadcast fertilization had a 40% smaller root mass fraction than unfertilized control plants while localized digestate depot fertilization and mineral NPK fertilization did not show a significant difference to unfertilized control plants.

#### Mesocosms

After three growing seasons in mesocosms, no significant difference between total biomass of plants fertilized with biogas digestate broadcast or depot fertilization was found (**Figure 3**). Depot fertilized plants developed a 25% larger root system than broadcast fertilized plants. NPK fertilized plants produced 20% less biomass than digestate-fertilized plants but six times more than the unfertilized control plants. Digestate depot fertilization resulted in nine times more biomass compared to the unfertilized control and broadcast digestate fertilization seven times, respectively.

### Leaf Nitrogen Content in Rhizotrons

In rhizotrons, the nitrogen concentration of the leaves for digestate broadcast and NPK fertilized plants was between 4 and 5% throughout the experimental time of 3 months (**Table 2**). After 30 days, digestate depot fertilized rhizotron plants only contained 2.9% of nitrogen in their leaves. After 60 days, nitrogen concentration increased to 4.1% and after 90 days it was in the same range as measured for digestate broadcast and NPK fertilized plants. Unfertilized control plants only contained 1.7% of nitrogen.

## Discussion

### Nitrogen Turnover in Rhizotrons

In accordance with the processes described of the CULTAN Method – proposed by Sommer (2003) for mineral ammonium fertilizers, the main nitrogen form of the digestate was mineralized from ammonium to nitrate. For digestate broadcast and NPK fertilization 80–90% of the measured nitrogen was available in the form of nitrate as early as 30 days after fertilization. Nitrate, compared to ammonium, is very mobile in the soil. Accordingly, nitrate was also the form of nitrogen that was found in the non-fertilized lower horizon of the rhizotrons. Since to the lower horizon, no nutrients were added, they must have arrived there by leaching (Baker, 2001). In rhizotrons receiving digestate depot fertilization, conversion from ammonium to nitrite was faster than the conversion from nitrite to nitrate. Accordingly, nitrite accumulated in a radius of 6 cm around the digestate depot. In digestate depot fertilized rhizotrons, digestate was applied locally in very high amounts and thus contained highest NH_4_^+^ concentrations. The combination of a regular watering of the rhizotrons with high concentration of organic material with a C/N-ratio of approx. 6 in the depot fertilized zone may have allowed for high microbial activity and created an environment with partially anoxic conditions that favors the formation of nitrite (Nelson and Bremner, 1969; Van Cleemput and Samater, 1995; Möller and Müller, 2012). In addition, the alkaline pH near the digestate fertilized zone favors the formation of nitrite in sandy soils (Greco et al., 2012). Once nitrite starts to accumulate in the soil, it can inhibit microbial activity. In a feedback loop, this may have further favored the formation of even more nitrite as transformation from ammonium to nitrite is not as much effected as the succeeding transformation from nitrite to nitrate (Grant et al., 1979). Nitrate, the mobile form of nitrogen was found from 12 cm distance to the digestate depot (Baker, 2001). Ammonium was only found up to a distance of 6 cm from the digestate depot, as it is not very mobile in soils (Clarke and Barley, 1968; Pang et al., 1973). In our study, 60 days after fertilization, ammonium was almost completely mineralized to nitrate, leaving only traces of ammonium and nitrite in digestate broadcast and NPK fertilized rhizotrons. In digestate depot fertilized rhizotrons, also after 60 days, high concentrations of ammonium were present, indicating a delayed conversion from ammonium to nitrate compared to broadcast fertilization (Grant et al., 1979; Sommer, 2005). Even though the high pH of >8 in the near depot zone was found to be very favorable for the mineralization of organic N-compounds from corn residues, like digestate (Deng and Tabatabai, 2000). The pH in the depot zone dropped to a range of pH 6.8–7.5 until day 60 after fertilization partially due to diffusion and partially due to the release of protons in the mineralization process (Giusquiani et al., 1995; Barak et al., 1997). Yet, also in digestate depot fertilized rhizotrons almost all ammonium was mineralized to nitrate within 90 days leaving only traces of nitrite and ammonium near the digestate depot.

### Nutrient Status and Root-Growth

#### Rhizotrons

**FIGURE 5:**
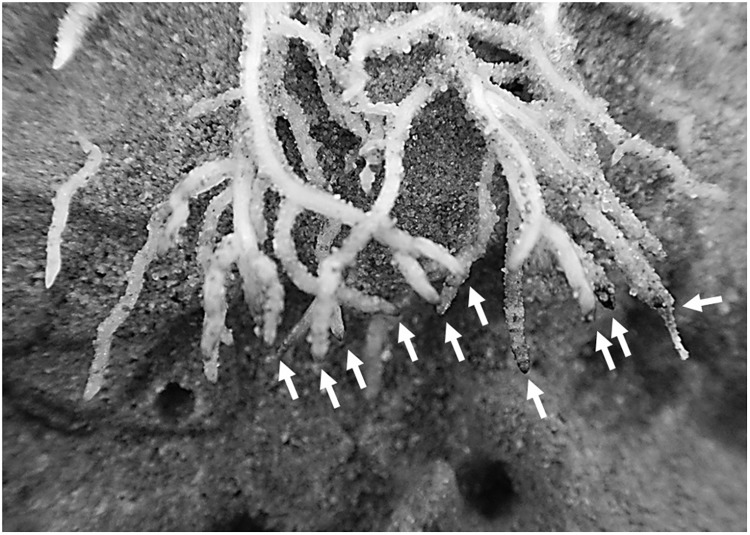
Brown and rotten root tips of *S. hermaphrodita* grown in digestate depot fertilized rhizotrons occurred in a 6 cm radius around the digestate depot 60 days after planting. Digestate depot fertilization was calculated to simulate a digestate application of 40 t ha^−1^. Arrows indicate the damaged root tips.

The root growth of *S. hermaphrodita* plants responded to the different fertilizer distributions and consequently different available forms of nitrogen over time. Even though there were no significant differences between fertilizer treatments in terms of biomass, clear differences in root distribution were observed (**Figures 2**, **3**). The broadcast application of digestate and NPK fertilizer, combined with a regular irrigation resulted in optimal supply of nutrients for seedlings of *S. hermaphrodita* (also indicated by the leaf nitrogen content >4%), reducing the necessity of seedlings to invest into root-growth (Forde and Lorenzo, 2001). Contrastingly, no nutrients were available in unfertilized control plants, while nutrients for digestate depot fertilized plants were not available in the rhizosphere (observable via the low leaf nitrogen content <3%). Consequently, plants invested into a deep and far-reaching root system to gain access to nutrients. These findings match the theory behind the CULTAN-method proposed by Sommer (2003) and also correspond well with results reviewed by Nkebiwe et al. (2016), focusing on controlled placement of fertilizers rich in ammonium. Additionally, the roots of plants growing in digestate depot fertilized rhizotrons, avoided to colonize a zone of approximately 6 cm radius from the digestate depot. This growing pattern was even more pronounced after 60 days. Here, roots in zones of the rhizotrons, not effected by fertilization, reached already the bottom of rhizotron while still very little root growth was observed in the 6 cm radius zone around the digestate depot. Given the high concentrations of ammonium and nitrite found within the 6 cm radius zone around the digestate depot in combination with root damages observed at root tips that contacted the area (**Figure 5**), we conclude that nitrite and ammonium toxicity are the main causes for the avoidance of this zone by the roots within the first 60 days after transplanting (Pan et al., 2016). Again, this temporarily zone of avoidance in a localized depot has been described earlier by Sommer (2003). Oke (1966) found that on sandy soil already nitrite concentrations >40 ppm can be toxic, especially to plant seedlings. In this experiment, we measured nitrite concentrations of <90 ppm in the depot zone. Further, ammonium toxicity was already studied intensively and observed when organic fertilizers, based on biogenic residues after anaerobic digestion were applied (Shaviv, 1988; Salminen and Rintala, 2002; Nkebiwe et al., 2016; Pan et al., 2016). Overall, results show consistently that high ammonium doses from digestate negatively affect seedling growth, mainly due to hampered root development.

**FIGURE 6:**
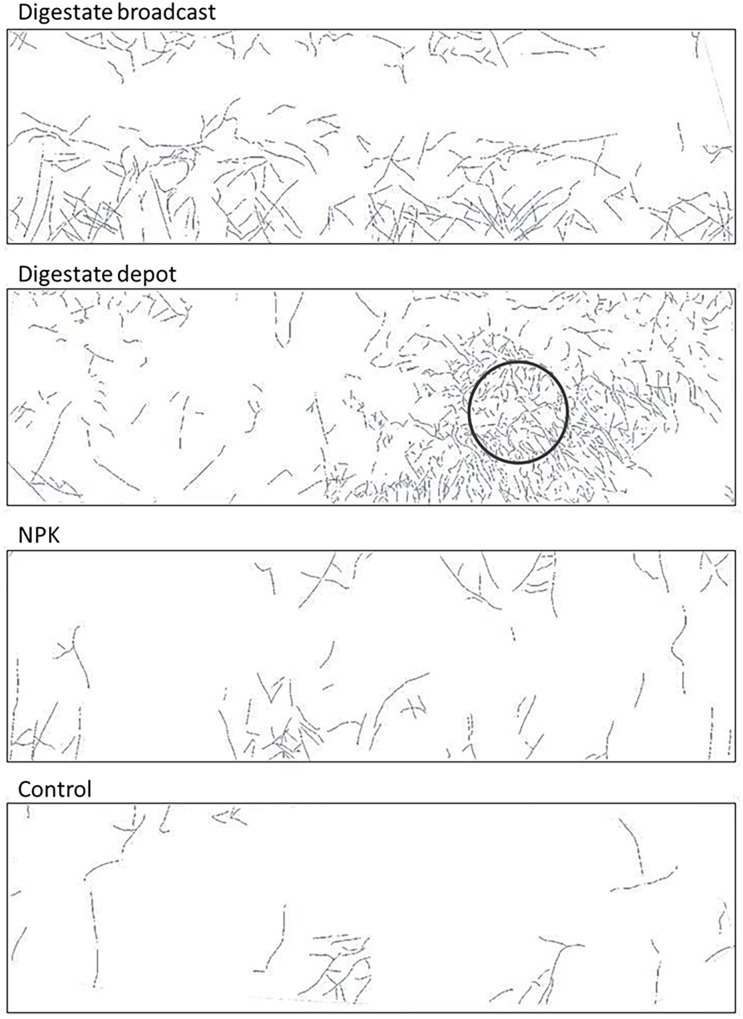
Root distribution of *S. hermaphrodita* cultivated in mesocosms measured by mini-rhizotrons after 2 years in 30 cm depth: roots form a dense root cluster in the depot-fertilized zone. Control: no fertilization; Digestate broadcast: homogeneous surface application; Digestate depot: localized digestate application; NPK homogeneous surface application. Pictures show a representative example of *n* = 4 replicates for each treatment and time of harvest.

After 60 days, the root growth pattern in depot-fertilized rhizotrons changed dramatically and 90 days after planting a dense root cluster of *S. hermaphrodita* around the nutrient rich digestate depot was observed. Root proliferation in nutrient rich patches was not observed yet for *S. hermaphrodita*, but for several herbaceous plant species (Rajaniemi and Reynolds, 2004). Plants can increase their rooting density in nutrient rich patches to forage for these nutrients, increasing the nutrient uptake efficiency and thus confer a competitive advantage toward weeds without access to nutrient patches (Robinson et al., 1999; Hodge, 2004; Rajaniemi, 2007). The increased access to nutrients is also shown by a parallel increase in leaf nitrogen content from only 2.9% at 60 days after transplanting to 4.4% measured in leaves of digestate depot fertilized *S. hermaphrodita* plants 90 days after transplanting. The massive increase of root biomass in the depot zone between day 60 and day 90 after planting, contributing to 50% of the total root mass of digestate depot fertilized *S. hermaphrodita* plants, resulted in the highest root mass across the four treatments (**Figure 1**). Also in our mesocosm study, the formation of dense root clusters in the digestate depot fertilized zone was observable throughout the 3-year experimental time under outdoor conditions (**Figure 6**).

In rhizotrons, after 90 days, NPK and digestate broadcast fertilization did not result in maximum possible rooting depth (i.e., depth of the rhizotron), and the major share of the root systems were located in the fertilized horizon. As plants were watered throughout the experiment, and roots had already sufficient access to the nutrients (indicated by the leaf nitrogen content >4%) plants did not invest into the formation of a deep reaching root system (Poorter et al., 2012b). Overall, the root systems that developed following digestate broadcast treatment remained smaller than the root systems of NPK fertilized plants. Phytotoxic effects of digestates, particularly affecting root growth of plants at early developmental stages, have been reported earlier and have besides ammonium toxicity also been explained by high concentrations of organic acids in fresh digestates (Salminen and Rintala, 2002; Abdullahi et al., 2008; Drennan and DiStefano, 2014). Control plants that had no access to nitrogen in the sandy substrate, resulting in a low leaf nitrogen content <2% and high investment into a deep reaching root-system, resulted in the highest root mass fraction of all variants (**Figure 4**) and thereby corresponded well to findings of previous studies (Poorter et al., 2012b).

#### Mesocosms

In mesocosms, the root mass fraction of *S. hermaphrodita* was generally higher after three growing seasons than found in rhizotrons. We explain this by the fact, that *S. hermaphrodita* is a perennial species, of which the shoots die off at the end of each vegetation period, while the major part of the root system stays intact and also serves as reservoir for a successful regrowth in the following growing period (Borkowska et al., 2009). As we only measured the root mass after three growing periods of mesocosms grown plants, this has a clear effect in the root mass fraction when compared to root mass measured after 90 days in rhizotrons.

### Biomass Yield

#### Rhizotrons

In rhizotrons, the above ground biomass increased gradually until day 60 of the experiment as plants invested mainly in root-growth. Accordingly, no significant differences in biomass of *S. hermaphrodita* were observed at day 60 throughout all treatments. This changed drastically when roots in digestate depot fertilized rhizotrons started to access the nutrient rich depot zone. The intense foraging for nutrients resulted in a faster increase in stem and leaf biomass as observed in all other variants. In his meta-analysis over 39 studies, Nkebiwe et al. (2016) also found a positive effect of localized fertilizer placement on biomass yield, especially for early plant development stages. Specifically for organic fertilizers like poultry litter (Pote et al., 2011), separated dairy sludge (Bittman et al., 2012) and manure slurries (Dell et al., 2000), the localized application had a positive effect on the total biomass.

Broadcast application of digestate yielded less biomass than NPK fertilization, even though leaf and soil analysis showed an adequate supply with nutrients and water for both variants. However, the root system of digestate broadcast fertilized plants was smaller and less outstretched as the root system of NPK fertilized plants as discussed earlier.

#### Mesocosms

After three vegetation periods of *S. hermaphrodita* in mesocosms under outdoor conditions, the different fertilization treatments showed different responses with respect to biomass than the same treatments in the rhizotrons. No significant biomass yield difference between digestate depot and digestate broadcast fertilization was observed. As discussed earlier, the localized placement of fertilizers has beneficial effects, especially in the early development stages (Nkebiwe et al., 2016). Further, the phytotoxic effects of the digestate, especially observed on the root growth of digestate broadcast fertilized plants in the rhizotrons, also relates to the early development stages of plants (Möller and Müller, 2012). Accordingly, it cannot be expected that effects last over this 3-year experimental time, especially as *S. hermaphrodita* is a perennial plant that keeps its extensive root system over the years (Borkowska et al., 2009).

The fact that digestate fertilization, independently from the type of application, produced higher biomass yields than mineral NPK fertilization, can be related to an increase of soil fertility over the years and has been discussed in a separate publication (Nabel et al., 2017).

### General Discussion

Localized application of biogas digestate as digestate depot in the rhizosphere of *S. hermaphrodita* seedlings fostered the successful establishment of *S. hermaphrodita* on marginal soils. Digestate depot fertilization strongly increased the rooting depth of seedlings, allowing improved access to water which could make them less susceptible to drought stress (Ma et al., 2009; Su et al., 2015). Drought can be the main growth-limiting factor, especially on marginal sandy soils with low WHC, making this deep rooting side effect of depot fertilization a positive one for adapting to drought regimes (Borkowska et al., 2009). The root-cluster formation around the digestate depot zone allowed the *S. hermaphrodita* seedling good access to the nutrients, resulting in rapid increase of biomass. Earlier studies showed that this can be an efficient way to make plants more competitive against weeds and thus lower the need for additional weed control (Blackshaw et al., 2002; Melander et al., 2005). Even though in the presented mesocosms study digestate depot fertilization did not show an increased biomass yield compared to digestate broadcast fertilization after three growing seasons, we still see a high potential in the application of digestate in the form of localized depots in the rhizosphere in marginal soils. As *S. hermaphrodita* is a perennial crop with an extensive root system, any form of soil cultivation in order to incorporate the digestate after a broad surface application via, e.g., plowing could do severe harm to the plants. In addition, a lack of digestate incorporation could cause high volatile losses of ammonia, with high environmental costs (Rochette et al., 2009; Ma et al., 2010). A controlled placement of digestate depots in the rhizosphere, e.g., via spike-weal injection, would minimize the impact on soil and also minimize volatile losses of ammonia (Kozlovský et al., 2009). As this application technique might also contribute to an increased competitiveness of *S. hermaphrodita* over weeds and thus reduce the need to weed control, it would further strengthen the concept of extensive biomass production on marginal soils.

## Conclusion

The application of digestate as a fertilizer in local depots into the rhizosphere of *S. hermaphrodita* grown in a marginal sandy substrate in rhizotrons and mesocosms resulted in a dense root formation around the depot-fertilized zone. After 3 months, half of the root biomass of depot-fertilized plants grown in rhizotrons was associated with the depot zone as a dense root-cluster; in contrast, root growth in the digestate broadcast fertilization treatment was strongly reduced. Overall, digestate depot fertilization resulted in a fivefold increase of total biomass compared to digestate broadcast fertilization in rhizotrons. Under outdoor conditions in a 3-year mesocosm experiment the increase in plant biomass was not significant, however. We conclude that digestate depot fertilization can contribute to an improved cultivation of *S. hermaphrodita* as a perennial energy-crop on marginal soils, especially for a successful establishment of seedlings, but its potential growth stimulation effect now needs more research under field conditions.

## Author Contributions

MN, SDS, HP, RK, and NDJ conceived the study. MN performed the main experiments and conducted the research under the supervision of SDS, HP, RK, and NDJ. MN wrote the manuscript. KN planned and designed *GrowScreen-Rhizo2*. CD and CB processed images and analyzed data. VT helped with study design and data evaluation. All authors discussed the results, assisted in the manuscript preparation, and contributed to revisions.

## Conflict of Interest Statement

The authors declare that the research was conducted in the absence of any commercial or financial relationships that could be construed as a potential conflict of interest.
